# 2111. Impact of Colonization by Multi Drug Resistant Bacteria on Graft Survival, Risk of Infection, and Mortality in Recipients of Solid Organ Transplant: Systematic Review and Meta-analysis

**DOI:** 10.1093/ofid/ofac492.1732

**Published:** 2022-12-15

**Authors:** Abdulellah Almohaya, Jordana H Fersovich, Benson Weyant, Oscar A Fernandez Garcia, Sandra M Campbell, Tamara Lotfi, Juan Gonzalez-Abraldes, Karen Doucette, Carlos Cervera, Dima Kabbani

**Affiliations:** University of Alberta, Edmonton, Alberta, Canada; University of Alberta, Edmonton, Alberta, Canada; University of Alberta, Edmonton, Alberta, Canada; University of Alberta, Edmonton, Alberta, Canada; University of Alberta, Edmonton, Alberta, Canada; McMaster University, Hamilton, Ontario, Canada; University of Alberta, Edmonton, Alberta, Canada; University of Alberta, Edmonton, Alberta, Canada; University of Alberta, Edmonton, Alberta, Canada; University of Alberta, Edmonton, Alberta, Canada

## Abstract

**Background:**

Colonization with multi-drug resistant bacteria (MDR) in solid organ transplant (SOT) recipients increases the risk of post-transplant bacterial infection. MDR colonization impact on graft survival and mortality is not well established.

**Methods:**

A search was executed by an expert librarian on PROSPERO, OVID Medline, Ovid EMBASE, Wiley Cochrane Library, ProQuest dissertations and Theses Global and SCOPUS, from inception until October 26, 2021. Adult SOT colonized with Methicillin resistant Staphylococcus aureus (MRSA), Vancomycin-resistant Enterococci (VRE), Extended-spectrum beta-lactamase (ESBL) or AmpC producing bacteria, carbapenem resistant Enterobacteriaceae (CRE), or MDR Pseudomonas were included and compared to non-colonized SOT. Pairs of reviewers screened abstracts and full studies for inclusion, and extracted data independently. We used RevMan to conduct a meta-analysis using random effects models to calculate the pooled risk ratio (RR) with 95% confidence interval (CI) for the incidence of infection, mortality, and graft failure. Statistical heterogeneity was determined using the I^2^ statistic.

**Figure-1:**
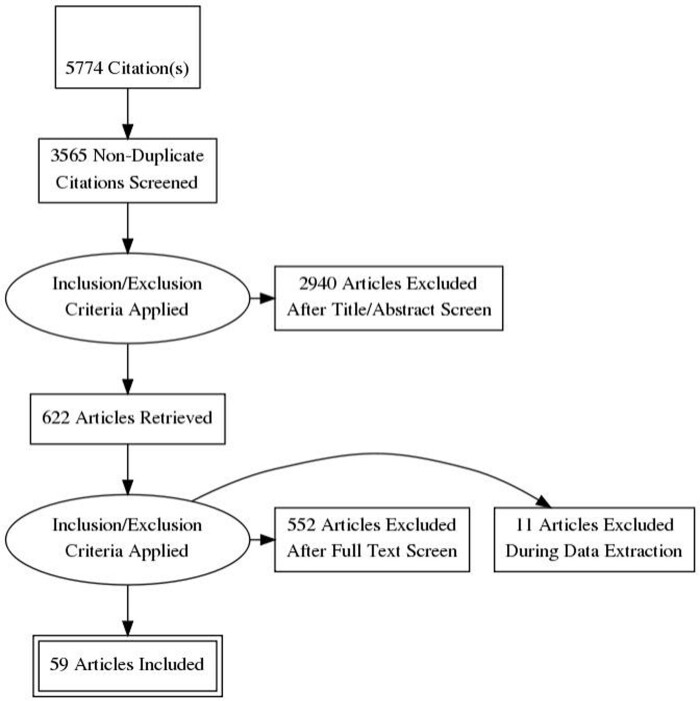

PRISMA chart, systemic review and metanalysis on Impact Of Colonization By Multi Drug Resistant Bacteria on Graft Survival, Risk of Infection, and Mortality in Recipients of Solid Organ Transplant.

**Results:**

59 articles spanning from 1989 to 2021 were included (Figure-1). Liver transplant (43 studies) and VRE colonization (17 studies) were the most common organ and MDR pathogen. MDR surveillance was performed by culture (71%) and PCR (6.7%). In liver transplant recipients, VRE and MRSA colonization were associated with increased infection risk, but not mortality (VRE infection: RR= 2.40 (95%CI 1.54-3.73; p< 0.001), I^2^= 66%; VRE mortality: RR= 1.64 (95%CI 0.88-3.05; p=0.12), I^2^= 44%; MRSA infection: RR= 4.07 (95%CI 2.66-6.24; p< 0.001), I^2^= 59%; MRSA mortality RR=1.47 (95%CI 0.79-2.76; p=0.23), I^2^= 35%). ESBL and CRE colonization were associated with increased risk of infection (ESBL: RR=9.87 (6.12-15.93); p< 0.001), I^2^=13%; CRE: RR= 13.64 (95%CI 5.73-32.47); p< 0.001), I^2^= 66%). CRE colonization was associated with increased mortality, RR=5.79 (95% CI 1.80-18.63; p=0.003), I^2^=0%.

**Conclusion:**

While colonization with MRSA and VRE in liver transplant was not associated with increase mortality, CRE colonization was associated with almost 6-fold increased risk of death. These data should be taken into account when stratifying the risk of transplant.

**Disclosures:**

**Carlos Cervera, Associate Professor**, Astra-Zeneca: Advisor/Consultant|AVIR Pharma: Grant/Research Support|AVIR Pharma: Honoraria|Lilly: Advisor/Consultant|Merck: Advisor/Consultant|Merck: Grant/Research Support|Merck: Honoraria|Sunovion: Advisor/Consultant|Takeda: Advisor/Consultant|Takeda: Honoraria|VerityPharma: Advisor/Consultant **Dima Kabbani, MD, MSc**, AVIR Pharma: Grant/Research Support|AVIR Pharma: Honoraria|GSK: Honoraria|Merck: Grant/Research Support.

